# Corticotropin-Releasing Hormone Stimulates Proopiomelanocortin Transcription via the CaMKK/CaMKIV Pathway in the AtT20 Cell Line

**DOI:** 10.7759/cureus.83257

**Published:** 2025-04-30

**Authors:** Wenyi Jiang, Haotian Zhang, Hitomi Imachi, Toshihiro Kobayashi, Kensaku Fukunaga, Takanobu Saheki, Takafumi Yoshimura, Tao Dong, Guoxing Zhang, Koji Murao

**Affiliations:** 1 Department of Endocrinology and Metabolism, Faculty of Medicine, Kagawa University, Kagawa, JPN; 2 Department of Endocrinology and Metabolism, Faculty of Medicine, kagawa university, Kagawa, JPN; 3 Department of Surgery, University of Michigan Health System, Ann Arbor, USA; 4 Department of Physiology and Neuroscience, Medical College of Soochow University, Suzhou, CHN

**Keywords:** adrenocorticotropic hormone, att20, camkk/camkiv pathway, corticotropin-releasing hormone, proopiomelanocortin

## Abstract

Proopiomelanocortin (POMC) is a critical precursor protein in the pituitary gland that regulates adrenal steroid hormone secretion by producing the adrenocorticotropic hormone (ACTH). Corticotropin-releasing hormone (CRH) modulates ACTH release via calcium influx through the voltage-operated Ca²⁺ channels and activation of Ca²⁺/calmodulin-dependent protein kinase II (CaMKII). In this study, we aimed to investigate the role of the calcium/calmodulin-dependent protein kinase kinase/ calcium/calmodulin-dependent protein kinase IV (CaMKK/CaMKIV) signaling cascade in CRH-induced POMC expression using ACTH-producing AtT20 cells, a cell line isolated from the pituitary gland of a mouse with tumor. Protein expression levels of CaMKK and CaMKIV were determined via western blotting. *POMC* transcription was analyzed via real-time polymerase chain reaction and reporter gene assays, and ACTH secretion was measured via enzyme-linked immunosorbent assay. In addition, effects of constitutively active CaMKK (CaMKK-CA) and CaMKIV (CaMKIV-CA) and their dominant-negative mutants on POMC promoter activity were assessed. CRH-induced CaMKIV phosphorylation was examined via western blotting. Both CaMKK and CaMKIV were expressed in the rat pituitary tissues; three random rats were used. Moreover, 10 nM CRH significantly increased *POMC* transcription and ACTH secretion in AtT20 cells. Inhibition of CaMKK and protein kinase A by STO-609 and H89, respectively, suppressed CRH-induced *POMC* transcription. Furthermore, CaMKK-CA and CaMKIV-CA independently activated the POMC promoter. CRH rapidly induced CaMKIV phosphorylation and nuclear localization, but these effects were blocked by STO-609. Overall, these findings suggest that the CaMKK/CaMKIV signaling pathway plays a crucial role in CRH-mediated *POMC* transcription and ACTH secretion in AtT20 cells.

## Introduction

Proopiomelanocortin (POMC) is a precursor protein that undergoes enzymatic cleavage to generate several biologically active peptides in pituitary corticotrophs, including the adrenocorticotropic hormone (ACTH), β-endorphin, and α-, β-, and γ-melanocyte-stimulating hormones. These peptides are involved in the regulation of key physiological processes, such as energy homeostasis, adrenal function, reproduction, thermoregulation, nociception, exocrine gland function, immune response, and pigmentation [1-3]. ACTH plays a crucial role in regulating adrenal steroidogenesis in response to stress signals [4,5].

POMC expression in pituitary corticotrophs is primarily controlled by hypothalamic corticotropin-releasing hormone (CRH) via CRH receptor 1 activation. This triggers multiple downstream signaling pathways, including the cAMP/protein kinase A (PKA) [6], mitogen-activated protein kinase (MAPK), and calcium signaling pathways, culminating in the transcriptional activation of POMC [7,8] by Nur protein family transcription factors [9]. These signaling pathways are also implicated in the modulation of other transcription factors, such as Tpit/PitxRE and activator protein 1, particularly via calcium-dependent mechanisms [10,11]. However, the direct role of calcium/calmodulin-dependent protein kinase IV (CaMKIV), a nuclear cAMP response element-binding protein (CREB) kinase [12], in CRH-induced POMC regulation remains unclear.

We previously demonstrated that the CaMKK/CaMKIV signaling cascade as critical for exendin-4-mediated gene transcription [13]. In this study, we aimed to further investigate whether the CaMKK/CaMKIV pathway similarly underlies CRH-induced POMC expression in ACTH-producing AtT20 pituitary tumor cells, broadening the understanding of calcium-dependent mechanisms in ACTH production.

## Materials and methods

Animals

Eight-week-old male SD (Sprague-Dawley) rats were maintained at room temperature, regulated on a 12-hour light/dark cycle. Rats were fed normal-salt chow and were allowed to access water freely. After four-week fed, three random rats were sacrificed and their pituitary tissues were removed, quickly frozen in liquid nitrogen and stored at -80 ℃. All experimental procedures involving animals were performed according to the Guidelines for the Care and Use of Animals as established by Kagawa University (approval no. 17646).

Cell culture

The murine corticotroph tumor cell line AtT20 was cultured in DMEM (Wako, Tokyo, Japan) containing 25 mmol/L glucose (high glucose type) supplemented with 10% heat-inactivated fetal bovine serum (Sigma, Tokyo, Japan), 100 U/mL penicillin, and 0.1 mg/mL streptomycin under 5% CO_2_/95% atmosphere at 37 ℃. Culture medium was changed twice a week, and the cells were subcultured once a week.

Real-time reverse transcription-polymerase chain reaction

Total cellular RNA was isolated with RNA-Bee-RNA isolation reagent and quantified by measuring the absorbance at 260 nm. Then, 6 μg of total RNA were used for reverse transcription. Mouse POMC gene was quantified by real-time PCR. The sequences of the forward and reverse mouse POMC primers were 5′-GATGCAAGCCAGCAGGTTGCTCTC-3′ and 5′-TGGAAGATGCCGAGATTCTGCTACAGT-3′. Glyceraldehyde-3-phosphate dehydrogenase (GAPDH) was used as housekeeping standard as previously described [13].

Western blot analysis

The 7.5% sodium dodecyl sulfate-polyacrylamide gel was used to separate the proteins, and then the proteins were transferred to a polyvinylidene difluoride membrane for immunoblotting. The membranes were blocked overnight at 4 degrees with 7.5% skimmed milk in PBS supplemented with 0.1% Tween 20. The blots were incubated overnight with anti-CaMKIV or anti-phosopho-Thr196-CaMKIV, anti-CaMKK α, or anti-TFIID primary antibody, followed by the incubation of an appropriate HRP-conjugated secondary antibody for one hour at 4 degrees. Membranes were again washed with PBS-T three times for 10 minutes each, and antigen-antibody complexes were visualized by ECL substrate (GE Healthcare). Protein bands in western blot analysis were obtained under Luminescent image analyzer LAS-1000 Plus (Fujifilm, Japan).

Enzyme-linked immunosorbent assay

Commercial ELISA kit was used for the determination of the ACTH release level in AtT20 cells (MD Bioproducts, Zurich, Switzerland). AtT20 cells were pretreated with inhibitors STO-609, H89, and LY294002, specific to CaMKK, PKA, and PI3K pathways separately. Following that, the cells were exposed to 10 nM CRH for 24 hours. The incubation media were then harvested, and ACTH levels in the supernatants were measured according to the manufacturer’s instructions.

Transfection and luciferase reporter assay

Subcloned chimeric construct contained the rat pomc genomic DNA and luciferase cDNA (pGL3-Basic, Promega, Madison, WI) were gifted from Yasumasa Iwasaki (Kochi university, Kochi, Japan). r-pomc-Luc was co-transfected with CaMKK/CaMKIV expressing plasmids or pcDNA into AtT20 cells at 70-80% confluence using lipofectamine (Life Technologies) according to the manufacturer’s instruction. Constitutively active CaMKK (CaMKIV-CA), dominant negative CaMKK (CaMKK-DN), constitutively active CaMKIV (CaMKIV-CA), and dominant negative CaMKIV (CaMKIV-DN) were constructed as described previously [14,15]. Transfected cells were maintained in media for 48 hours. pcDNA and CaMKK-DN/CaMKIV-DN transfected cells were exposed to 10 nM CRH for additional 24 hours. Then, AtT20 cells were harvested, and the luminescence of luciferase level was measured according to the manufacturer’s instructions (Toyo Ink, Tokyo, Japan).

Statistical analysis

Data were expressed as mean ± standard error of mean (SEM). The results were analyzed using one-way ANOVA and student’s t test by GraphPad Prism (version 8.0.2.263, GraphPad Software, Inc. LLC, Boston, MA, USA), statistical significance was presented as ^*^P < 0.05 in all figures. All experiments were performed in triplicates at least.

## Results

Expression levels of CaMKIV and CaMKK in the pituitary tissues

**Figure 1:**
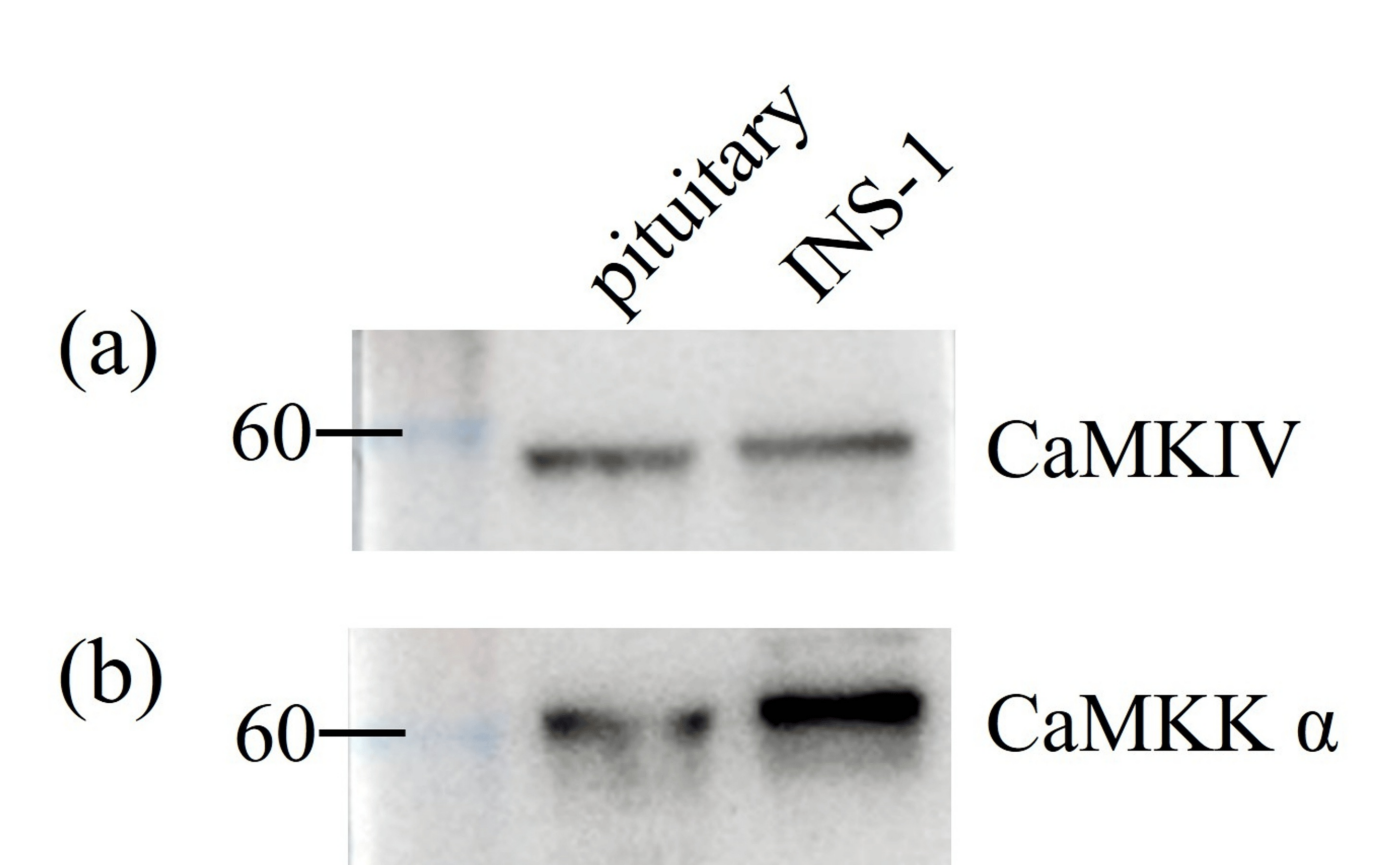
Expression levels of calmodulin-dependent protein kinase (CaMK)-IV and CaMKKα in rat pituitary tissues. (a) CaMKIV levels. (b) CaMKKα levels. Protein levels in INS-1 cells were used as positive controls.

First, CaMKIV and CaMKKα protein levels were determined in rodent pituitary tissues (Fig. 1). INS-1 cell line, a rat insulinoma-derived cell line, was used as a positive control for protein expression analysis.

Effect of the CaMKK/CaMKIV cascade on CRH-induced POMC transcription in AtT20 cells

**Figure 2:**
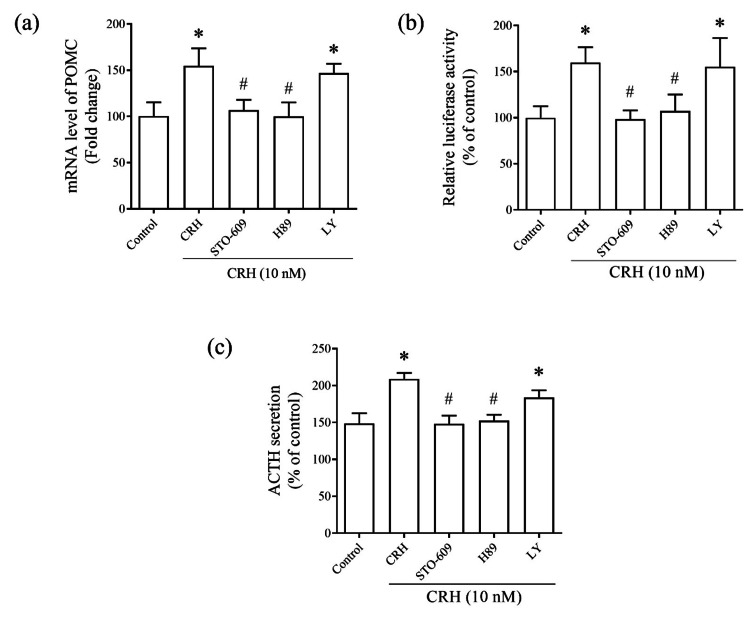
Roles of the protein kinase A (PKA)/CaMKK cascade in corticotropin-releasing hormone (CRH)-induced proopiomelanocortin (POMC) transcriptional activity and adrenocorticotropic hormone (ACTH) secretion in AtT20 cells. Effects of CaMKK inhibitor STO-609, PKA inhibitor H89, phosphatidylinositol 3-kinase (PI3K) inhibitor LY294002, or no treatment (control) on CRH (10 nM)-induced (a) POMC mRNA levels, (b) POMC promoter activity, and (c) ACTH secretion. Data were normalized to the control group data. Data are represented as the mean ± standard error of the mean (SEM). ^*^P < 0.05 vs. control; ^#^P < 0.05 vs. CRH derived from ordinary one-way ANOVA, followed by Tukey’s multiple-comparison test and Student’s t-test.

CRH plays key roles in stimulating POMC gene transcription and ACTH secretion [16]. To investigate the roles of protein kinases in CRH-induced POMC transcription, we assessed the effects of pharmacological inhibitors on POMC mRNA levels and promoter activity and ACTH secretion. AtT20 cells were treated with CRH (10 nM) in combination with the CaMKK inhibitor STO-609 (1 μM), PKA inhibitor H89 (1 μM), or phosphatidylinositol 3-kinase inhibitor LY294002 (10 μM). Notably, phosphatidylinositol 3-kinase inhibitor LY294002 had no effect, whereas both STO-609 and H89 significantly inhibited CRH-induced POMC transcription (Fig. 2a, 2b). A similar inhibition pattern was observed for ACTH secretion (Fig. 2c), suggesting that the PKA/CaMKK/CaMKIV signaling pathway is involved in CRH-mediated POMC transcription and ACTH secretion.

Regulation of POMC promoter activity by the CaMKK/CaMKIV pathway in AtT20 cells

**Figure 3:**
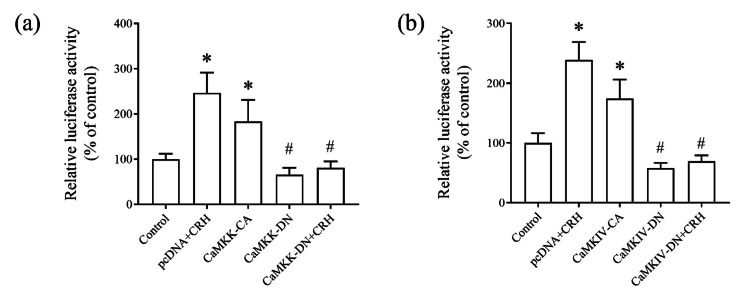
Effect of the CaMKK/CaMKIV cascade on the POMC promoter activity. AtT20 cells were co-transfected with the rat POMC promoter-luciferase construct (r-pomc-Luc) or empty vector plasmid cloning DNA (pcDNA) and expression plasmids of (a) CaMKK-constitutively active (CA), CaMKK-dominant-negative (DN), (b) CaMKIV-CA, or CaMKIV-DN for 48 hours. Then, pcDNA- and CaMKK-DN/CaMKIV-DN-transfected cells were exposed to 10 nM CRH for 24 hours. The total luciferase activity was measured. Data were normalized to the control group data. Data are represented as the mean ± SEM. ^*^P < 0.05 vs. control; ^#^P < 0.05 vs. pcDNA + CRH derived from ordinary one-way ANOVA, followed by Tukey’s multiple-comparison test.

To further examine the role of the CaMKK/CaMKIV pathway in CRH-mediated POMC regulation, we co-transfected AtT20 cells with a rat POMC promoter-luciferase construct (r-pomc-Luc) with either constitutively active (CA) or dominant-negative forms (DN) of CaMKK and CaMKIV or an empty plasmid cloning DNA (pcDNA) vector (control). Cells transfected with the dominant-negative forms of CaMKK and CaMKIV were treated with CRH for 24 houra. CRH stimulation significantly increased the POMC promoter activity, which was further enhanced by the constitutively active forms of CaMKK and CaMKIV. Conversely, dominant-negative forms of CaMKK and CaMKIV abolished the CRH-induced activation of POMC promoter activity (Fig. 3a, 3b). These findings suggest that the CaMKK/CaMKIV signaling cascade plays a critical role in the CRH-mediated regulation of POMC gene expression.

Time course of CRH-induced CaMKIV phosphorylation in AtT20 cells

**Figure 4:**
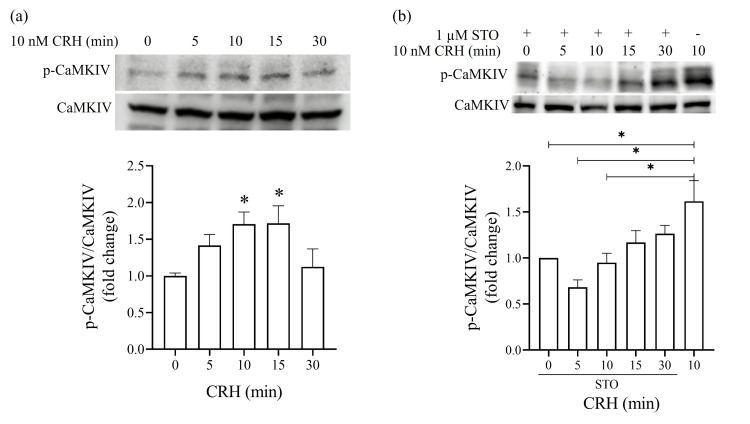
Time course of CaMKIV phosphorylation by CRH in AtT20 cells. (a) CRH stimulates CaMKIV phosphorylation. The cells were exposed to 10 nM CRH for 0, 5, 10, 15, and 30 minutes. High phosphorylated CaMKIV levels were determined via western blotting analysis of the total cell proteins using the phospho-Thr196-CaMKIV antibody. The p-CaMKIV/CaMKIV ratio is shown as a percentage of the basal ratio. Data were normalized to the control group data. (b) CRH stimulates CaMKIV phosphorylation via the CaMKK signaling pathway. The cells were treated with the CAMKK inhibitor STO-609 and exposed to 10 nM CRH for 0, 5, 10, 15, and 30 minutes. Cells treated with CRH alone for 10 minutes were used as positive controls. The p-CaMKIV/CaMKIV ratio is shown as a percentage of the basal ratio. Data were normalized to the control group data. Data are represented as the mean ± SEM. ^*^P < 0.05 derived from ordinary one-way ANOVA, followed by Dunnett’s multiple-comparison test.

To confirm the role of CaMKIV in CRH-induced POMC transcription, we assessed the time course of CaMKIV phosphorylation following CRH stimulation via western blotting analysis. The phosphorylation of CaMKIV at Thr196, a site targeted by CaMKK, was examined using a phospho-Thr196-specific monoclonal antibody. CRH treatment rapidly increased CaMKIV phosphorylation, with significant activation observed within 15 minutes (Fig. 4a).

To determine whether CRH-induced CaMKIV phosphorylation occurs via the CaMKK signaling pathway, we pre-treated AtT20 cells with the CaMKK inhibitor STO-609 (1 μM) prior to CRH stimulation. Notably, the CaMKK pathway inhibition completely blocked the CRH-induced CaMKIV phosphorylation (Fig. 4b), further supporting the role of the CaMKK/CaMKIV cascade in CRH-mediated POMC transcription.

## Discussion

In this study, we demonstrated that both CaMKK and CaMKIV were expressed in the rat pituitary tissues. Moreover, CRH-induced ACTH secretion was suppressed by PKA and CaMKK inhibitors in AtT20 cells. In corticotrophs of pituitary tissues, CRH binds to the CRH receptor 1, stimulating the production and release of ACTH into the systemic circulation, which subsequently induces the synthesis of glucocorticoids, including cortisol. Excessive cortisol production, driven by supraphysiological levels of ACTH, leads to Cushing’s disease, which is mainly characterized by obesity, hypertension, and diabetes [17,18]. Surgical resection is the primary treatment for Cushing’s disease, and various medical therapeutics, including steroidogenesis inhibitors, corticotroph-directed agents, and glucocorticoid receptor blockers, are often used to inhibit ACTH production and prevent cortisol overproduction. Notably, the suppression of CRH-induced ACTH secretion by CaMKK inhibitors in vitro highlights the therapeutic potential of targeting CaMKK/CaMKIV pathway in Cushing’s disease.

CRH is a key regulator of ACTH secretion that stimulates POMC transcription and ACTH release in pituitary corticotrophs [1,3]. Our findings are consistent with a prior report that the CaMKK/CaMKIV signaling cascade plays an essential role in anterior pituitary hormone regulation, particularly in thyrotropin-releasing hormone signaling [19]. Here, CaMKK-CA and CaMKIV-CA independently stimulated POMC promoter activity, whereas CaMKK-DN and CaMKIV-DN prevented CRH-induced POMC transcription, supporting the role of the CaMKK/CaMKIV pathway in CRH-mediated POMC gene regulation.

However, this study focused on in vitro models, AtT20 cells, which limits the understanding of the physiological relevance of CaMKK/CaMKIV in intact organisms. While in vitro system provided critical mechanistic insights, future investigations in vivo, such as pituitary-specific CaMKIV-knockout or CaMKIV-overexpression mice that can be generated to study ACTH secretion phenotypes, are essential to validate the CaMKK/CaMKIV pathway’s role in ACTH secretion under systemic hormonal and neuronal regulation.

Multifunctional CaMK family, including CaMKI, CaMKII, and CaMKIV, is regulated via CaMKK phosphorylation and involved in various cellular processes, such as muscle contraction, neurotransmitter release, and gene expression [20]. Unlike CaMKII, which is activated via autophosphorylation, CaMKIV promotes neuronal survival by activating CREB in the nucleus [21,22]. Previous studies have shown that CaMKIV mediates transcription via CREB phosphorylation [23,24]. consistent with our previous findings that the co-transfection of CaMKK-CA and CaMKIV-CA significantly enhances CREB-mediated transcription [25]. CaMKK-induced CaMKIV activation markedly increases CREB phosphorylation [26], suggesting the critical role of the CaMKK/CaMKIV pathway in transcriptional regulation.

Despite the absence of a CREB-binding site in the POMC promoter, CREB, a PKA-dependent phosphorylation target, is expressed in corticotrophs and possibly mediates CRH-induced ACTH secretion via POMC transcription [27]. In this study, we found that CRH stimulated CaMKIV phosphorylation at Thr196 via the CaMKK pathway, thereby increasing POMC transcription and ACTH secretion. These results suggest CaMKIV phosphorylation as a key mediator of CRH stimulatory effect on ACTH production.

CRH activates multiple transcription factors involved in POMC transcription, including Nur factors, Tpit/PitxRE, NeuroD1, CRH-responsive element-binding protein, CREB, AP-1, and nuclear factor-κB, via cAMP/PKA-induced CaMKII activation [1]. CRH-induced transcriptional activities of Nur and Tpit/PitxRE are regulated via PKA-induced Ca2+ influx that activates CaMKII [7]. Consistently, this study also revealed that PKA was involved in the regulation of POMC promoter activity by CRH. CRH-induced activation of Tpit/PitxRE transcriptional activity is attenuated by L-type calcium channel inhibitors and CaMKII blockers [11]. Intracellular calcium mediates CRH-induced expression of immediate early c-Fos via calcium-binding protein DREAM, which relieves the repression of the c-Fos gene promoter [28]. Through its role in c-Fos induction, CREB forms a heterodimer with Jun and functions as an AP-1 transcription factor activating POMC transcription [29]. Despite the absence of a CREB-binding element in the POMC promoter, CREB acts as its co-activator at the NurRE site [3,30].

## Conclusions

This study demonstrated that the CaMKK/CaMKIV signaling cascade was involved in the CRH-induced POMC gene transcription and ACTH secretion in AtT20 cells. Our findings suggest that this pathway plays a crucial role in the regulation of anterior pituitary function, particularly in response to CRH stimulation. Further studies are needed to elucidate the precise mechanisms by which CaMKK/CaMKIV affects POMC transcription in corticotrophs.
